# CHROMOPHOBE HEPATOCELLULAR CARCINOMA: DIAGNOSTIC CHALLENGES

**DOI:** 10.1590/0102-6720202400069e1863

**Published:** 2025-01-20

**Authors:** Sana Ben-Slama, Ines Mallek, Eya Ghorbeli, Mohamed Hajri, Taher Labidi, Hafedh Mestiri, Ahlem Lahmar, Dhouha Bacha

**Affiliations:** 1 Mongi Slim Hospital, Department of Pathology – Marsa, Tuni, Tunísia; 2 University of Tunis, Faculty of Medicine of Tunis, – El Manar, Tunis, Tunísia; 3 Salah Azaiez Institute, Department of Oncologic Surgery, Tunis, Tunísia; 4 Mongi Slim Hospital, Department of General Surgery, Tunis, Tunísia.

**Keywords:** Pathology, Carcinoma, Hepatocellular, Liver Diseases, Tumor Cells, Cultured, Patologia, Carcinoma Hepatocelular, Hepatopatias, Células Tumorais Cultivadas

## Abstract

**BACKGROUND:**

Hepatocellular carcinoma (HCC) encompasses rare variants like chromophobe hepatocellular carcinoma (CHCC) characterized by distinct histological features and molecular profiles.

**CASE REPORT:**

A 56-year-old male with chronic hepatitis C, presenting pain in the right hypochondrium. Imaging revealed a solitary liver lesion, subsequently resected and histologically diagnosed as HCC. Macroscopic examination found a 4×4 cm encapsulated liver nodule with necrotic areas, surrounded by numerous smaller satellite nodules in Segment 6. The liver was in micronodular cirrhosis. Histologically, the tumor had focal trabecular or pseudoglandular patterns within a vascularized stroma. The cells were large, with clear to eosinophilic cytoplasm and hyperchromatic and pleomorphic nuclei with focal anaplastic features. No vascular invasion was noted in adjacent cirrhotic liver tissue.

**RESULTS:**

The final diagnosis was CHCC. Due to its rarity and overlapping characteristics with other hepatic tumors, CHCC poses diagnostic challenges. Accurate diagnosis necessitates thorough histopathological assessment and molecular testing. The identification of the alternative lengthening of telomeres phenotype may distinguish CHCC from conventional HCC and hold potential implications for targeted therapeutic approaches.

**CONCLUSIONS:**

Recognition of HCC variants is critical for effective management and underscores the need for continued research into its clinical behavior and therapeutic responses.

## RESUMO

**RACIONAL:**

O carcinoma hepatocelular (CHC) abrange variantes raras como o carcinoma hepatocelular cromófobo (CHCC) caracterizado por características histológicas e perfis moleculares distintos.

**RELATO DE CASO:**

Masculino, de 56 anos com hepatite C, apresentando dor no hipocôndrio direito. Exames de imagem revelaram uma lesão hepática solitária, posteriormente ressecada e histologicamente diagnosticada como CHC. O exame macroscópico encontrou um nódulo hepático encapsulado de 4x4 cm com áreas necróticas, cercado por vários nódulos satélites menores, no Segmento 6. O fígado estava em cirrose micronodular. Histologicamente, o tumor tinha padrões trabeculares ou pseudoglandulares focais dentro de um estroma vascularizado. As células eram grandes, com citoplasma claro a eosinofílico e núcleos hipercromáticos e pleomórficos com características anaplásicas focais. Nenhuma invasão vascular foi observada no tecido hepático cirrótico adjacente.

**RESULTADOS:**

O diagnóstico final foi um CHCC. Devido à sua raridade e características sobrepostas com outros tumores hepáticos, o CHCC apresenta desafios diagnósticos. O diagnóstico preciso necessita de avaliação histopatológica completa e testes moleculares. A identificação do fenótipo ALT pode distinguir o CHCC do HCC convencional e tem implicações potenciais para abordagens terapêuticas direcionadas.

**CONCLUSÕES:**

O reconhecimento de variantes de CHCs é crítico para o gerenciamento eficaz e ressalta a necessidade de pesquisa contínua sobre seu comportamento clínico e respostas terapêuticas.

## INTRODUCTION

Hepatocellular carcinoma (HCC) is a common primary liver cancer^3,4^. According to the 2019 World Health Organization classification, up to 35% of HCCs exhibit subtypes defined by histological and molecular characteristics^12^.

Chromophobe hepatocellular carcinoma (CHCC) is a rare variant characterized by unique cellular morphology and staining properties^14^. At first, described by Wood et al.^15^, CHCC features include chromophobic cytoplasm and pronounced nuclear pleomorphism with bland nuclei and scattered microscopic pseudocysts^15^. CHCC, occurring in up to 3% of cases, presents with clear cytoplasm and focal nuclear atypia within an otherwise "bland" cytological background.

This subtype is notably associated with the alternative lengthening of telomeres (ALT), a telomerase-independent mechanism sustained via homology-directed repair^7^. Molecularly, most cases demonstrate an ALT phenotype via telomere fluorescence in situ hybridization (FISH)^15^.

The prognosis for CHCC is similar to conventional HCC^13^. Due to its rarity, CHCC often poses diagnostic challenges and can be mistaken for other hepatic or non-hepatic malignancies.

This case report emphasizes the importance of recognizing and accurately diagnosing this rare entity for effective management.

## CASE REPORT

This is a 56-year-old patient who has been monitored since 2016 for hepatitis C virus-induced cirrhosis, classified as CHILD B with a MELD (Model for End-Stage Liver Disease) score of 9. The condition has been complicated by four episodes of digestive hemorrhage caused by grade II esophageal varices rupture, which were managed with symptomatic treatment.

**Figure 1:**
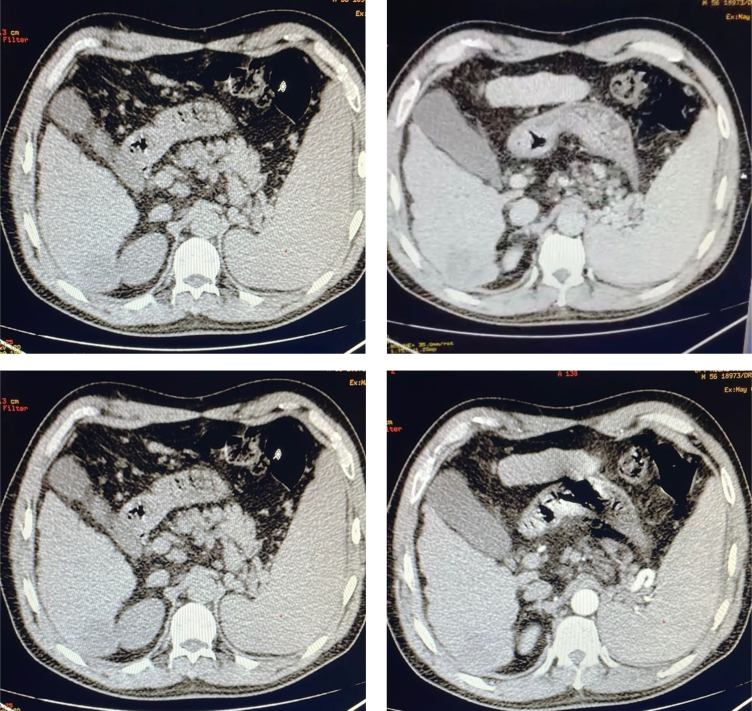
The CT scan images at various arterial and venous phases: a dysmorphic liver with cirrhosis and a spontaneously hypodense lesion in segment VI, measuring 44×33 mm, with heterogeneous enhancement in the arterial phase and homogenized in the venous phase.

The patient presented pain in the right hypochondrium. He was anicteric and the abdominal examination revealed no abnormalities. The biological hepatic panel was normal with no evidence of biological inflammatory syndrome and no elevation of AFP (alpha-fetoprotein) rate. An abdominal ultrasound was requested. It showed a hypotrophic liver with overall heterogeneous cirrhosis presenting a 32×30 mm heterogeneous echogenic nodular lesion in segment VI, associated with homogeneous splenomegaly. A thoraco-abdominal-pelvic computed tomography (CT) scan was performed. It revealed a dysmorphic liver with cirrhosis and a spontaneously hypodense lesion in segment VI measuring 44×33 mm. This lesion showed heterogeneous enhancement in the arterial phase and homogenized in the venous phase, strongly suggesting HCC in a cirrhotic liver (Figure 1). Additionally, no secondary localization was noted.

## RESULTS

The patient underwent surgery via a right subcostal approach, during which an atypical resection of the nodule in segment VI and a cholecystectomy were performed.

**Figure 2:**
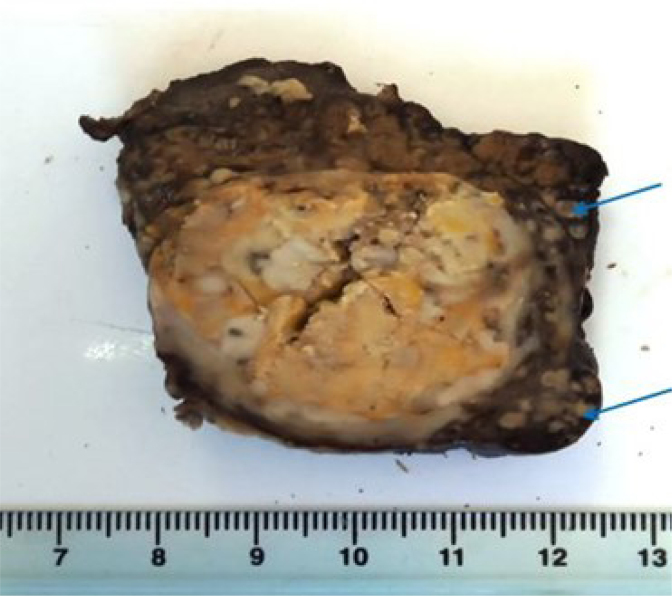
Macroscopic examination: a well-circumscribed, encapsulated nodule measuring 4×4 cm with a mottled white-yellowish appearance. Numerous satellite nodules (<2 cm in diameter and located within 2 cm of the primary tumor) were present (blue arrows).

**Figure 3:**
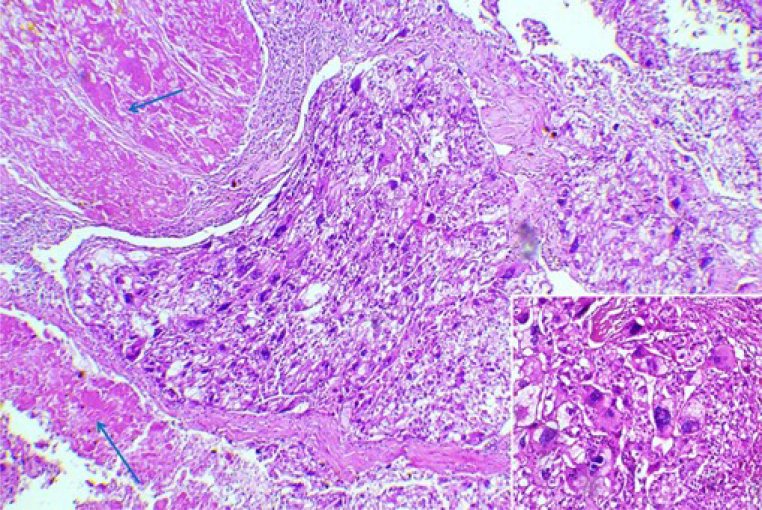
Microscopic examination: carcinomatous proliferation with focal necrotic changes (blue arrows) (hematoxylin-eosin ×100). Cartridge: tumor cells were large, round, or polygonal, some with eosinophilic and granular cytoplasm. The nuclei showed multiple anaplastic features with a few abnormal mitoses (hematoxylin-eosin ×200).

On macroscopic examination, the hepatic resection measured 10×6×4 cm with a micronodular cirrhotic liver. On sectioning, a 4×4 cm well-circumscribed, encapsulated subcapsular nodule with a mottled white-yellowish and necrotic appearance was found, located 1 cm from the nearest surgical margin. Numerous satellite nodules were present (Figure 2). Histological examination showed carcinomatous proliferation with focal trabecular or pseudoglandular patterns. Focal necrotic changes were noted within a finely vascularized stroma. The tumor cells were round or polygonal, mostly clear, with some eosinophilic, granular cytoplasm. The nuclei were very hyperchromatic, irregular, pleomorphic, and occasionally showed multiple anaplastic features, with a few abnormal mitoses (Figure 3). Satellite nodules were confirmed as carcinomatous foci. No vascular emboli or endobiliary extensions were observed. The surgical resection margin was clear, with a minimum healthy margin of 10 mm. The cirrhosis was mildly active, and the gallbladder was safe.

## DISCUSSION

CHCC is a rare variant of the HCC a rare subtype, accounting for approximately 3% of HCC cases^12^.

Combining our case, the total number of relevant cases studied across these sources amounts to 37^8,15^. It is characterized by tumor cells with clear to pale eosinophilic cytoplasm and generally mild nuclear changes. However, some cells exhibit significant nuclear pleomorphism^11^. These unique histological features underscore the importance of accurate differentiation for appropriate treatment. Despite these peculiarities, CHCC shares the same prognosis as a common HCC^12^.

Early identification and surgical intervention can significantly improve patient outcomes^9^.

An important study involving 219 HCC cases at Johns Hopkins University was accomplished by Wood et al. between 1985 and 2011. Two liver pathologists reviewed these cases, identifying 13 with distinct histological features suggestive of a novel HCC subtype^15^. According to their results, no typical male predominance was observed in this subtype compared to typical HCCs^15^.

The population similarly demonstrated a tendency toward a higher prevalence of CHCC among females, though this trend did not reach statistical significance (54% vs. 34%, p=0.061, p>0.05)^15^. In a Korean study^8^, which identified 23 cases of CHCC among 224 cases, significant differences were noted, with a higher prevalence in females (p=0.023, p>0.05). The patient in our case is a 56-year-old male, aligning with the typical gender distribution seen in HCC.

Background liver conditions within this cohort at Johns Hopkins University varied: six cases exhibited cirrhosis, one case had bridging fibrosis, one case had portal fibrosis, and five cases showed no fibrosis^15^. Among the 13 cases identified in this cohort, there was a notable association with hepatitis B virus (HBV), which occurred more frequently compared to typical HCCs. CHCC was more prevalent in HBV carriers compared to conventional HCC (46% vs. 16%, p=0.006, p<0.05)^15^. Chronic viral hepatitis was observed in seven cases, six linked to HBV and one to hepatitis C virus (HCV). Additionally, one case was associated with alcoholic cirrhosis, another with cryptogenic cirrhosis, and one presented as a hepatic adenoma with fatty change. Furthermore, one case had no known underlying liver disease, while two cases lacked sufficient clinical information for detailed characterization^15^. In our case, the patient has an active hepatitis B viral infection, a significant risk factor for HCC. There is no significant history of alcohol consumption or other liver disease risk factors reported.

HCC often develops in patients with liver cirrhosis, primarily due to viral hepatitis or chronic alcoholism. It can remain asymptomatic until an advanced stage, complicating early diagnosis^3^. HCC can exhibit significant biological and genetic diversity^12^.

Preoperative AFP level is a marker for assessing the progression and severity of HCCs^15^. For the patient, the AFP levels were within the normal range.

Wood et al. documented imaging findings. Magnetic resonance imaging (MRI) and CT scans were performed^15^. The scans revealed that the tumors were hypervascular in the arterial phase and exhibited washout in the portal venous phase, characteristic of HCC. One case showed hypervascularity in the arterial phase, progressive enhancement in the portal venous phase, and large areas of central necrosis. On MRI, the tumors appeared slightly hypointense on T1-weighted and slightly hyperintense on T2-weighted images, without intra- or extracellular fat. Most of the tumors exhibited a thick pseudocapsule with no evidence of lymphadenopathy. In our case, contrast-enhanced MRI characterized the lesion as a heterogeneous mass with a capsule-like rim, which is suggestive of a primary liver tumor, typical of HCC.

Several pathology variations of the CHCC were noted. Tumor size averaged 5.4±3.8 cm, indicating a moderate size range. Pseudocapsules were present in most of the cases, often characterized by thick and irregular formations, contributing to the tumor's encapsulation^15^.

Histologically, CHCC features tumor cells with smooth chromophobic cytoplasm, marked nuclear pleomorphism with abrupt anaplasia^8^ and a background of cells with bland nuclei, along with scattered microscopic pseudocysts^1^.

Intratumoral fibrosis was identified frequently, which contributed to the tumor's structural complexity. Angiolymphatic invasion occurred in rare cases, highlighting potential aggressive behavior. Geographic tumor necrosis was noted, with a history of prior embolic therapy, suggesting a possible treatment-related effect.

Additionally, a case exhibited a cholestatic appearance, and several cases showed varying degrees of steatosis, with some having minimal macrovesicular steatosis and others showing mild macrovesicular steatosis^15^. In our case, the tumor with focal trabecular or pseudoglandular patterns, exhibited necrotic carcinomatous proliferation within a finely vascularized stroma. Tumor cells were mostly clear, round, or polygonal, with some cells showing eosinophilic, granular cytoplasm. The nuclei were hyperchromatic, irregular, and pleomorphic, with anaplastic features, abnormal mitoses.

Concerning molecular characteristics, currently, HCCs with abrupt anaplasia lack a definitive immunostain for confirmation. However, telomere-specific FISH for the alternative lengthening of telomeres (ALT) phenotype could potentially fulfill this role^5^.

CHCC is notably linked with the ALT, a telomerase-independent mechanism facilitating telomere length maintenance without (telomerase reverse transcriptase — TERT) promoter mutations or other TERT gene rearrangements^6^.

Prior studies have demonstrated a high prevalence of ALT in CHCC (92% of cases studied), contrasting sharply with only 8% of unselected HCC cases being ALT-positive. Nevertheless, beyond ALT, comprehensive clinicopathological and molecular characteristics of CHCC remain poorly elucidated^7^.

The differential diagnosis of CHCC encompasses various conditions as a hepatocellular adenoma when the cytoplasmic is fat and glycogenic but the cells have uniform nuclei and low nuclear-cytoplasmic ratio^2,10^. A combined hepatocellular-cholangiocarcinoma can be discussed but lacks the scattered foci of nuclear anaplasia observed in CHCC^2^. The main differential diagnosis is metastatic chromophobe renal cell carcinoma, identifiable by large pale cells with prominent cell membranes and perinuclear haloes, but often expressing CK7 (cytokeratin 7) and CD117 (c-kit) immunopositivity^2^.

On the other hand, the clear cell HCC variant presents another challenging differential, especially in cases with patchy areas of anaplasia. Clear cell HCC typically exhibits distinct nuclear features outside of anaplastic regions, contrasting with the cytologically bland nuclei and inconspicuous nucleoli characteristic of chromophobe variants. Variations in cytoplasmic staining properties can assist in distinguishing between these entities^10,15^.

Treatment and prognosis for HCC involve a range of approaches depending on the stage and patient's liver function, including surgical resection, liver transplantation, and locoregional therapies like transarterial chemoembolization or ablation^12^. CHCC, a rare variant, lacks standardized treatment guidelines but generally involves individualized approaches. The prognosis of this condition is similar to classical HCC. However, outcomes still depend on factors such as tumor size, stage, and response to treatment^15^. Recent clinical trials evaluating targeted chemotherapeutic agents in unselected HCC have yielded disappointing results, highlighting the critical need to identify patient subgroups more likely to benefit from specific agents or combinations^9^. The reliance on ALT via homologous recombination suggests promising opportunities for targeted therapeutic approaches in the management of CHCC^15^.

## CONCLUSION

CHCC, despite its rarity, should be considered in the differential diagnosis of hepatic tumors displaying atypical histological features. First, this subtype represents a distinctive morphological classification within HCC. Then, there is an expectation that these tumors may exhibit unique gene-expression patterns compared to typical HCCs, which is crucial for identifying biomarkers and developing targeted therapies.

Finally, CHCCs with abrupt anaplasia may demonstrate differential responses to standard treatments compared to typical HCCs, emphasizing the need for tailored therapeutic strategies. Effective management needs multidisciplinary collaboration to ensure accurate diagnosis and personalized treatment plans.

Central Message Hepatocellular carcinoma (HCC) is a common primary liver cancer, and according to the World Health Organization classification of 2019, up to 35% of HCC cases have subtypes with specific histological and molecular profiles. Among these, chromophobe hepatocellular carcinoma is a rare variant defined by unique cellular morphology and staining characteristics, which can make diagnosis challenging and increase the likelihood of misidentification as other hepatic or non-hepatic malignancies.

Perspectives Despite its rarity, chromophobe hepatocellular carcinoma (CHCC) should be included in the differential diagnosis of hepatic tumors with atypical histological features. This subtype represents a unique morphological classification within hepatocellular carcinoma and is expected to have distinct gene expression profiles, which are critical for identifying biomarkers and developing targeted therapies. The management of CHCC benefits from multidisciplinary collaboration to achieve precise diagnosis and personalized treatment, underscoring the importance of further research into its clinical characteristics and treatment responses.
